# Transgenerational Effects of Hexavalent Chromium on Marine Medaka (*Oryzias melastigma*) Reveal Complex Transgenerational Adaptation in Offspring

**DOI:** 10.3390/biom11020138

**Published:** 2021-01-22

**Authors:** Xiaomin Ni, Yingjia Shen

**Affiliations:** 1 Key Laboratory of the Coastal and Wetland Ecosystems, Ministry of Education, Xiamen University, Xiamen 361005, China; xiaominni@stu.xmu.edu.cn; 2 School of Life Science, Fudan University, Shanghai 200433, China; 3 Fujian Key Laboratory of Coastal Pollution Prevention and Control, Xiamen University, Xiamen 361102, China

**Keywords:** *Oryzias melastigma*, hexavalent chromium, DNA, transgenerational effects, environmental adaptation

## Abstract

Hexavalent chromium [Cr(VI)] pollution is one of most serious heavy metal pollutants in the coastal area and posed serious threats to marine organisms and human beings. Many studies have been conducted on its toxicological effects on living organisms from morphological to physiological aspects. However, there are few studies about the transgenerational toxicological of Cr(VI). In this study, we exposed adult marine medaka fishes with Cr(VI) and their offspring with Cr(VI) to examine transgenerational effects of Cr(VI). We found that there were mechanisms such as changing reproduction modes in males to compensate for impacts on the reproduction. There were differences and similarities between the parental effect and the environmental effect, with the former one causing more serious adverse effects on the offspring of Cr(VI)-exposed fish. It was noteworthy that there was an interaction between the parental and offspring treatment which leads to the attenuation of the parental effects on offspring when the offspring also underwent the same treatment. In addition, physiological adaptation has also been observed in fish to improve their fitness. Overall, effects of Cr(VI) on fish and their offspring were studied to pave a way to study the of mechanisms of adaptation.

## 1. Introduction

Chromium (Cr) and its compounds are important industrial materials widely used in metallurgy, leather processing, pulp production, mining, etc., [1,2]. With the development of industrialization in coastal areas, chromium pollution seriously increased [3]. Generally, the soluble Cr content in water was 10–500 ng/L, but the dissolved Cr content in some industrial areas had exceeded 1 mg/L. For example, the highest concentration of Cr in coastal waters could reached 2.4 mg/L in Yancheng. [4]. Serious chromium pollution posed potential threats on marine organisms and human beings. Hexavalent chromium was the main type of Cr produced by anthropogenic activity, with high toxicity, high solubility, and high mobility [5].

Early life stages of fish from fertilization to early juvenile stage were thought as the most sensitive period to pollutants. Embryo and juvenile fish were commonly used to study pollutant toxicity directly, but embryos were often parentally exposed and directly exposed to pollutants in developmental environments. Toxicity transfer from parental exposure, including pollutant delivery, would have a serious impact on the development of the embryo [6]. For example, Vignet et al. found that after exposure to polycyclic aromatic hydrocarbons (PAHs), even if the offspring was under an unexposed environment with no detected PAHs, the offspring still suffered from the same behavioral interferences as the parents [7]. Recent research shows that heavy metals could be accumulated in fish, resulting in developmental malformations, delayed growth, and mortality [8]. Cr(VI) was found to induce pathological changes in the liver of marine medaka including nuclear migration, cell vacuolization, etc., [9]. The influence of environmental pollutants on organisms are also found to be passed on to the next generation, so the transgenerational effects of environmental pollution should be an important part of ecological risk assessment [10]. So far, there have been few studies about transgenerational effects of Cr(VI).

In this study, marine medaka (*Oryzias melastigma*), a model species for marine toxicological studies, was used as the experimental animal. We exposed adult marine medaka to Cr(VI) and explored the transgenerational effects of Cr(VI) on offspring from daily changes to long-term observation. In addition, we exposed offspring to Cr(VI) to evaluate the transgenerational effects of Cr(VI) vs. developmental exposures. Overall, transgenerational effects of pollutants were studied in fish, which could provide the basis for ecological evolution of Cr(VI) and improvement of traditional toxicological studies.

## 2. Materials and Methods

### 2.1. Experience Fishes

Marine medaka used in the study were provided by Coastal State Key Laboratory of Marine Environmental Science in Xiamen University, and cultured under a constant condition (28‰ salinity, 28 °C, a photoperiod of 14h-light:10h-dark).

### 2.2. Experimental Design

Five-month-old adult fishes with similar body size were chosen for the exposure experiment. Sodium chromate (Na_2_CrO_4_, Sigma-Aldrich, St. Louis, MO, USA) was used as the exposure substance. The exposure concentration used in this study was based on the 96h-LC50. There were three replicates, with each replicate containing one male and one female in a 350 mL plastic tank (PE level). Before exposure, experimental fishes were exposed for two weeks; exposure containers were soaked in 10% nitric acid overnight and washed twice using distilled water used as a rinsing agent. The half-static renewal treating method (replace half of liquid in the tank every 24 h) was used as the treating method [11,12].

**Figure 1:**
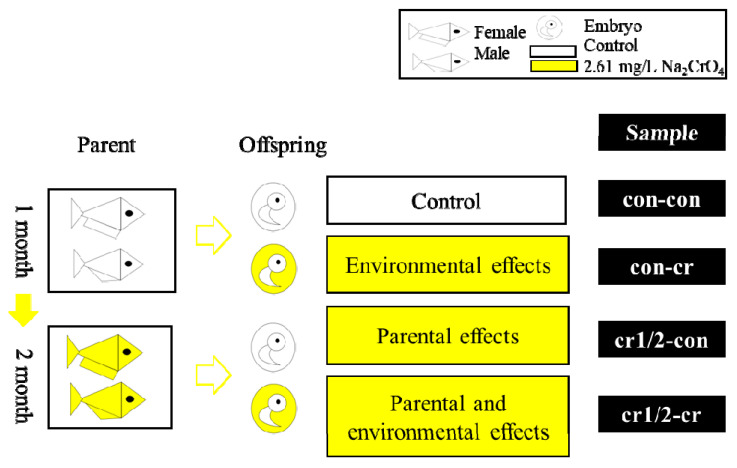
Diagram of experimental design for studying transgenerational effects of Cr(VI) of marine medaka. White represent control environment, yellow represents Cr(VI) treatment; there were four kinds of treatment, represented by “parental treatment-offspring treatment”, i.e., con-con, con-cr, cr1/2-con and cr1/2-cr; cr1 -con and cr1-cr represent treatments of the first month of parental treatment, cr2-con and cr2-cr represent treatments of the second month of parental treatment; con-cr was used to study developmental exposures, cr1/2-con was used to study parental effects, and cr1/2-cr was used to study combined effects of developmental exposures and parental effects.

We cultured the adult fishes in a controlled environment (28‰ salinity, 28 °C, a photoperiod of 14 h-light:10h-dark) for one month and exposure for two months. During the experiment, eggs were collected at 9 am every day, and cultured in a control or Cr(VI) environment. We defined parental effects as effects that come from parents while environmental effects are defined as effects that come from the external environment except the parental environment. A detailed exposure strategy is shown in the Table 1 and Figure 1. Fish and their offspring were examined every day. We observed and recorded situation of spawning eggs, fertilization, deformation, mortality, heart rate, and hatch under microscope (SMZ-168, Motic Europe, Barcelona, Spain). All animal experiments follow the guidelines of the Fujian Provincial Department of Science and Technology on the management of laboratory animal affairs.

### 2.3. Parameter Calculation

#### 2.3.1. Parental Fecundity

We defined the egg laying amount in a certain time as female fecundity and fertilization rate as an assessment of male fecundity. We determined that eggs that enter the cleavage stage within two hours after the collection were fertilized eggs [13]. The rate of fertilization was calculated as follows:

fertilization rate (%) = fertilized eggs number/total eggs number × 100%(1)

#### 2.3.2. Death Rate and Malformation Rate

We determined an embryo without a heartbeat or movement as a dead embryo. Death rate was calculated as follows:

death rate (%) = dead embryos number/total embryos number × 100%(2)

The embryo with visible malformation under microscope was regarded as malformed embryo. The calculation of malformation rate was as follows:

malformation rate (%) = abnormal embryo number/total embryo number × 100%(3)

#### 2.3.3. Heart Rate

We recorded the heart rates at 5 d, 8 d, and 11 d after fertilization. 5 d embryo formed regular heartbeat, 8 d embryo reached highest heartbeat, and 11 d embryo achieved constant heartbeat [14]. We counted the heartbeat times in 30 s and repeated for three times. The heart rate per minute was calculated as follows [15]:

heart rate = heartbeat times per 30 s × 2(4)

#### 2.3.4. Hatch Situation

The hatch rate was calculated as follows:

hatching rate (%) = hatched embryo number/total embryo number × 100%(5)

The hatch time was defined as the time from fertilization to complete hatch.

Usually, the normal hatch time of the marine medaka embryo was 14 d. If hatch time was longer than 14 d, the embryo developed slowly [16], which we defined as hatch delay. Hatch delay rate and hatch delay time is calculated as follows:

hatching delay rate (%) = the number of embryos hatched/the total number of embryos × 100%(6)

hatch delay time (d) = hatch time − 14(7)

### 2.4. Statistical Analysis

All experimental data in this experiment are expressed as mean ± SD. All data were analyzed by SPSS v20.0.0 software. One-way ANOVA with Tukey HSD and Paired Sample T test is used for comparisons between different Cr(VI) concentrations or generations. Two-way ANOVA were used when both Cr(VI) concentrations and generational effects are considered. Confidence level *p* < 0.05 was significantly different, the confidence level of *p* < 0.01 was extremely significantly different.

## 3. Results

### 3.1. Cr(VI) Reduced Maternal Fecundity

**Figure 2:**
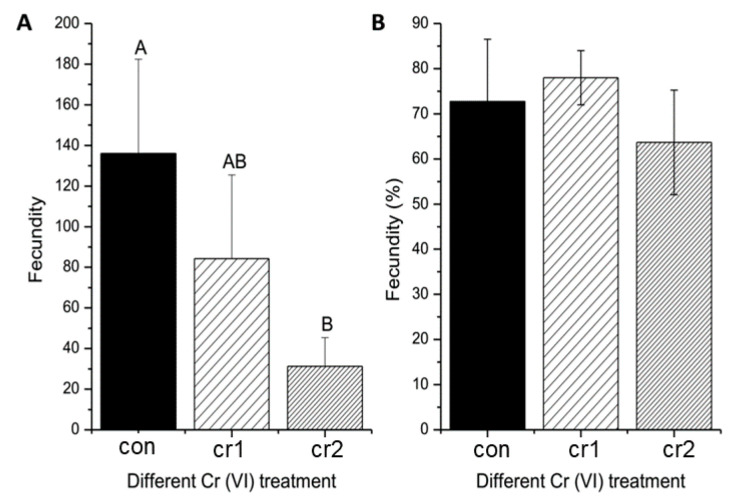
The change of parental reproduction induced by long-term Cr(VI) exposure on marine medaka. (**A**) the change of female fecundity, different upper case means the significant differences among different treatment (*p* ≤ 0.01); (**B**) the change of fertilization rate. For different treatment group, the number of biological samples is 4.

After long-term exposure of Cr(VI), we found that the amount of spawning decreased, and the amount of spawning in the second month was significantly lower than those before exposure (see Figure 2A), similar to the studies for Japanese medaka [17]. Although Cr(VI) exposure had resulted in a slight decrease of fertilization rate, the fertilization rate has not been significantly affected (see Figure 2B). The unaffected fertilization rate was also found in toxicological studies of other heavy metal such as Zn and Cu [18].

### 3.2. Cr(VI) Led to Increase of Death Rate

**Figure 3:**
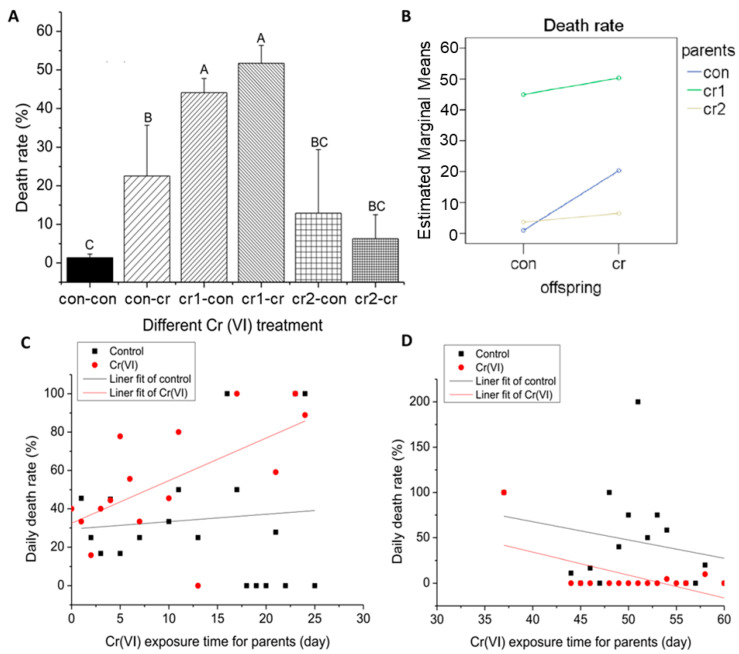
The change of death rate after long-term Cr(VI) exposure in marine medaka offspring. (**A**) the death rate of offspring under different Cr(VI) treatment, different upper case means the significant differences among different treatment (*p* ≤ 0.001); (**B**) the interaction of parent and offspring Cr(VI) treatment for death rate (*p* = 0.000); (**C**,**D**) daily death rate under different offspring Cr(VI) exposure after parental Cr(VI) exposure, X axis represents parental treatment, and Y axis represents daily death rate, the blank line represents offspring in control environment, the red line represents offspring in Cr(VI) environment; (**C**) parental Cr(VI) exposure for first month, the function relationships between exposure time and death rate were y = 0.3858x + 29.42182, R^2^ = −0.04964 (untreated offspring) and y = 2.22099x + 32.4783, R^2^ = 0.31578 (treated offspring); (**D**) parental Cr(VI) exposure for second month, the function relationships between exposure time and death rate were y = −2.01x + 148.4, R^2^ = −0.01 (untreated offspring) and y = −2.51x + 134.7, R^2^ = 0.292 (treated offspring). The number of biological samples used for each group is as follow: con-con (*N* = 486), con-cr (*N* = 81), cr1-con (*N* = 145), cr1-cr (*N* = 159), cr2-con (*N* = 90), cr2-cr (*N* = 87).

We found that Cr(VI) treatment leads to an increase in death rate (see Figure 3A,B), which is the same as Cr(VI) toxicological studies in other fish [19]. For the first month of parental treatment, death rate of offspring in control environment (cr-con1) was significantly higher than that of only offspring treated by Cr(VI) (con-cr). The death rate for the second month of parental treatment (cr2-con/cr2-cr) was significantly lower than that for the first month of parental treatment (cr1-con/cr1-cr). There was a significant interaction between the parental treatment and offspring treatment on the death rate.

Daily death rate was different for the first month and second month of parental Cr(VI) treatment (see Figure 3C,D). For the first month, the offspring under Cr(VI) treatment showed an increased death rate compared to offspring without treatment, and the difference of death rate between the two groups was increased. For the second month, the daily death rate of offspring under Cr(VI) treatment was obviously lower than that of offspring without treatment.

### 3.3. Cr(VI) Was Highly Teratogenic

**Figure 4:**
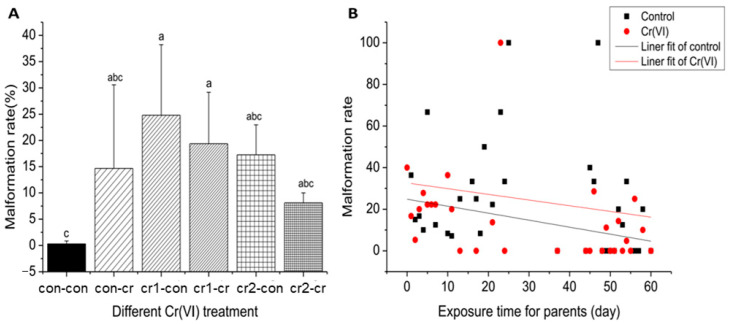
The malformation rate after long-term Cr(VI) exposure (**A**) the malformation rate of offspring under different Cr(VI) treatment, different lower case means the significant differences among different treatment (*p* ≤ 0.05); (**B**) daily malformation rate under different Cr(VI) exposure after parental Cr(VI) exposure, X axis represents parental treatment, and Y axis represents daily malformation rate, the blank line represents offspring in control environment, the red line represents offspring in Cr(VI) environment; the function relationships between exposure time and death rate were y = −0.3x + 24.8, R^2^ = 0.10 (untreated offspring) and y = −0.2x + 32.6, R^2^ = 0.01 (treated offspring). The number of biological samples used for each group is as follow: con-con (*N* = 486), con-cr (*N* = 81), cr1-con (*N* = 145), cr1-cr (*N* = 159), cr2-con (*N* = 90, cr2-cr (*N* = 87).

Malformation during development is a sensitive index commonly used in embryo experiments. We observed the development of embryos, and evaluated the abnormalities of the embryo according to the published experimental procedures [20]. The change in malformation rate under different treatments was similar to the death rate (see Figure 4A). The malformation rate ranged from 8% to 25%, such high malformation rate as also observed by other published studies [16,21]. After parental treatment, daily malformation rate of offspring under Cr(VI) treatment stayed higher than offspring without treatment (see Figure 4B).

**Figure 5:**
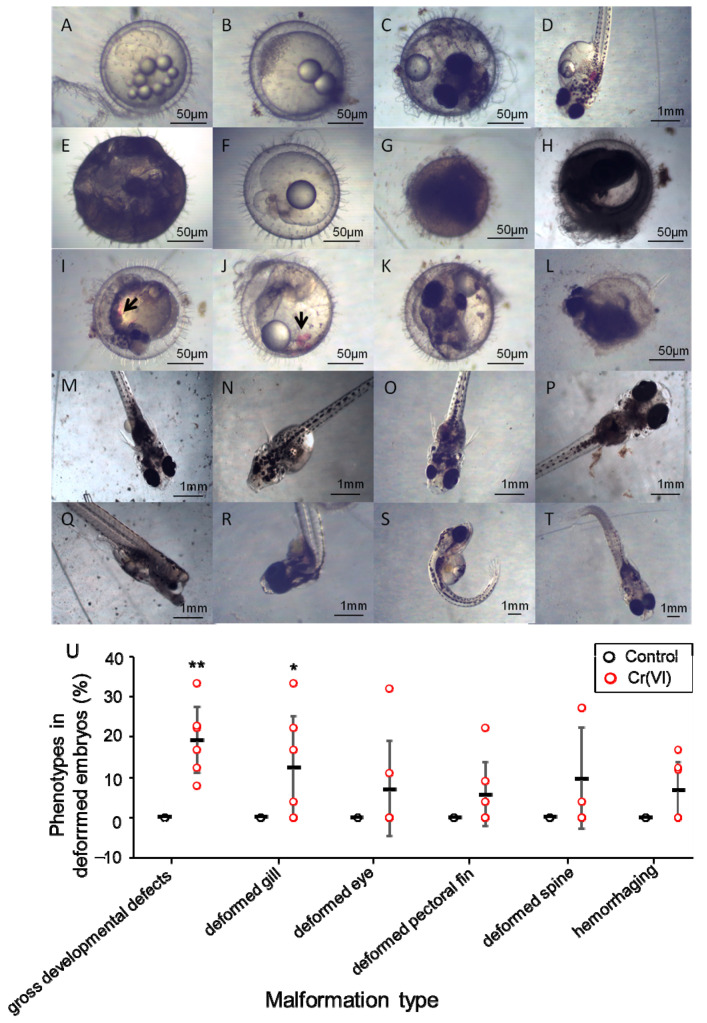
Abnormalities in F1 generation of marine medaka with Cr(VI) exposure. (**A**–**D**) Normal embryo and larvae; (**E**) seriously stained unfertilized egg; (**F**) embryo with abnormal blastoderm; (**G**) stained embryo; (**H**) dead embryo; (**I**) embryo with gross developmental defects and hemorrhaging; (**J**) eyeless embryo and hemorrhaging; (**K**) embryo with abnormally shaped eye; (**L**) embryo hatched unsuccessfully; (**M**) larvae with small yolk sac; (**N**) deformed larvae with abnormally shaped eye and single pectoral fin; (**O**) deformed larvae with abnormally shaped eye;(**P**) deformed larvae without pectoral fin; (**Q**) deformed larvae with abnormal head; (**R**–**T**), deformed larvae with curled spine); Arrowheads, hemorrhaging; (**U**) the malformation rate of common malformations; **, *p* ≤ 0.01; *, *p* ≤ 0.05. The number of biological samples used for each group is as follow: con-con (*N* = 486), con-cr (*N* = 81), cr1-con (*N* = 145), cr1-cr (*N* = 159), cr2-con (*N* = 90), cr2-cr (*N* = 87). The number of biological samples used for each group is as follow: con (*N* = 652), cr (*N* = 746).

We recorded the types of deformed embryo (see Figure 5A–T). We found that the rate of gross developmental defects (19.24%) and gill malformation (12.70%) after Cr(VI) treatment were highest, followed by spinal deformities (9.76%), malformation of eyes (7.19%), partial hemorrhage (6.86%), and malformation of pectoral fins (5.89%).

### 3.4. Parental Cr(VI) Treatment Delayed Heat Development

**Figure 6:**
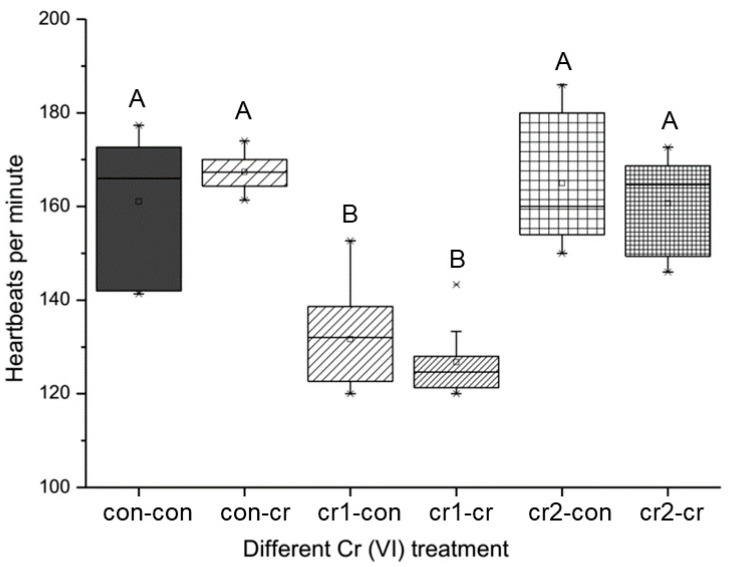
The change of overall heartbeat after long-term Cr(VI) exposure in marine medaka offspring. Different upper case means the significant differences among different treatment (*p* ≤ 0.001). The number of biological samples used for each group is as follows: con-con (*N* = 14), con-cr (*N* = 25), cr1-con (*N* = 9), cr1-cr (*N* = 9), cr2-con (*N* = 3), cr2-cr (*N* = 3).

The heart was one of the first functional organs during embryonic development, so it is the most sensitive to environmental pollutants. We found that Cr(VI) treatment on the offspring (con-cr) led to increased heart rate, while the parental Cr(VI) treatment significantly reduced the heart rate for the first month (cr1-con&cr1-cr), and the heart rate would be reduced much more when the offspring were also treated by Cr(VI) (cr1-cr) (see Figure 6). It is worth noting that when the parental Cr(VI) was treated for two months (cr2-con&cr2-cr), the heart rate of offspring would return to relative normal levels.

**Figure 7:**
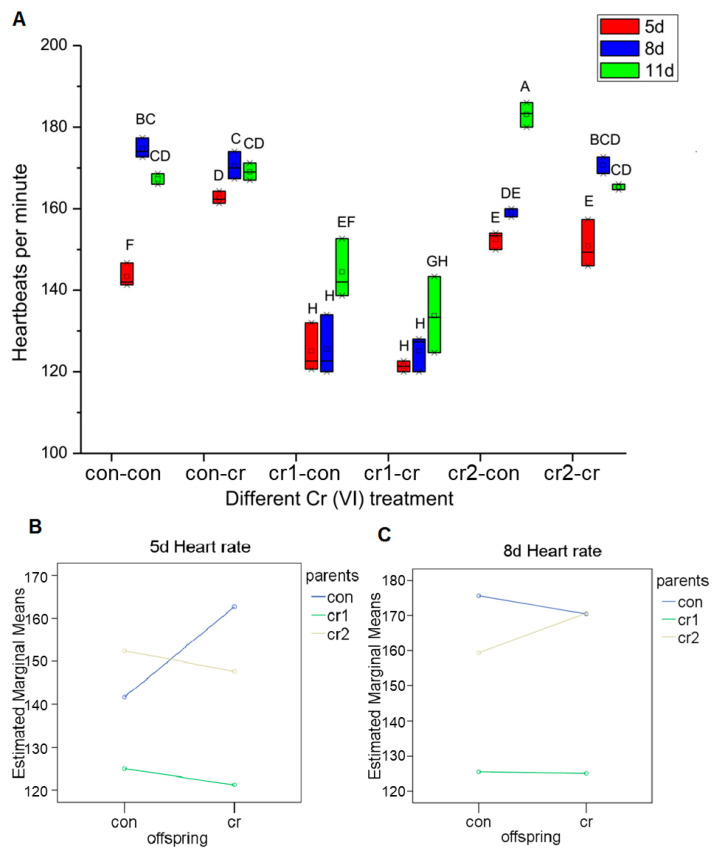
The change of heart rate after long-term Cr(VI) exposure in marine medaka offspring. (**A**) the heart rate for 5 d, 8 d and 11 day of offspring under different Cr(VI) treatment, different upper case means the significant differences among different treatment (*p* ≤ 0.001); (**B**,**C**) the interaction of parent and offspring Cr(VI) treatment for heart rate; (**B**) 5 d heart rate (*p* = 0.000); (**C**) 8 d heart rate (*p* = 0.031). The number of biological samples used for each group is as follow: con-con (*N* = 14), con-cr (*N* = 25), cr1-con (*N* = 9), cr1-cr (*N* = 9), cr2-con (*N* = 3), cr2-cr (*N* = 3).

For different stages of heart rates (see Figure 7A), we found that the developmental model of the offspring treatment was similar to that of the control, but the heart rate of 5 d in offspring treatment (con-cr) increased significantly. The developmental model of the different parental treatment was extremely different. For the first month of parental treatment (cr1-con&cr1-cr), it would obviously decrease heart rate during development process, especially for 5 d and 8 d. For the second month of parental treatment (cr2-con&cr2-cr), the heart rate of the offspring in the Cr(VI) environment (cr2-cr) was not significantly different from the normal embryo, but the heart rate of the 8 d in the blank environment (cr2-con) was significantly lower than that of a normal embryo (con-con), and the heart rate of the 11 d (cr2-con) was significantly higher than the maximum heart rate of a normal embryo. We also found that there was an extremely significant interaction between the parental treatment and offspring treatment on 5 d and 8 d heart rate (see Figure 7B,C).

### 3.5. Parental Cr(VI) Treatment Delayed Hatch Process

#### 3.5.1. Parental Cr(VI) Treatment Reduced Hatch Rate

**Figure 8:**
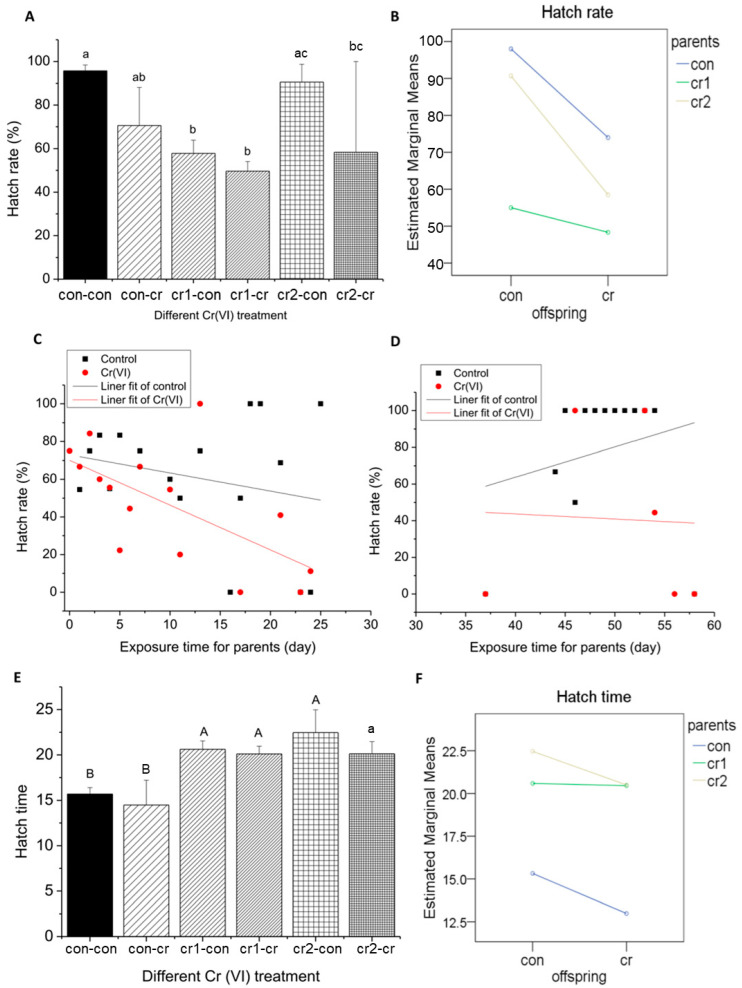
The hatch situation after long-term Cr(VI) exposure in marine medaka offspring. (**A**–**D**) hatch rate; (**A**) the hatch rate of offspring under different Cr(VI) treatment, different lower case means the significant differences among different treatment (*p* ≤ 0.05); (**B**) the interaction of parent and offspring Cr(VI) treatment for hatch rate (*p* = 0.030); (**C**,**D**) daily hatch rate under different offspring Cr(VI) exposure after parental Cr(VI) exposure, X axis represents parental treatment, and Y axis represents daily hatch rate, the blank line represents offspring in control environment, the red line represents offspring in Cr(VI) environment; (**C**) parental Cr(VI) exposure for first month, the function relationships between exposure time and death rate were y = −0.96x + 72.98, R^2^ = −0.006 (untreated offspring) and y = −2.38x + 70.07, R^2^ = 0.352 (treated offspring); (**D**) parental Cr(VI) exposure for second month, the function relationship between exposure time and hatch rate were y = 1.64903x − 2.2166, R^2^ = −0.003403 (untreated offspring) and y = −0.27718x + 54.78429, R^2^ = −0.24754 (treated offspring); (**E**,**F**), hatch time; (**E**) the hatch time of offspring under different Cr(VI) treatment, different letter means the significant differences among different treatment (upper letter: *p* ≤ 0.01, lower letter: *p* ≤ 0.05); F, the interaction of parent and offspring Cr(VI) treatment for hatch time(*p* = 0.001). The number of biological samples used for each group is as follow: con-con (*N*= 315), con-cr (*N* = 55), cr1-con (*N* = 120), cr1-cr (*N* = 141), cr2-con (*N* = 37), cr2-cr (*N* = 21).

The effects of Cr(VI) on hatch rate after different treatments were similar to the mortality rate, malformation rate, and heart rate (see Figure 8A). Offspring treatment (con-cr) would decrease hatch rate slightly, which coincides with the study on zebrafish [22]. In parental treatment, hatch rate increased for the first month (cr1-con&cr1-cr), but restored to relative high level for the second month (cr2-con&cr2-cr). When parents and offspring were treated (cr1-cr& cr2-cr), hatch rate would reach to the lowest value.

After parental exposure, daily hatch rate of offspring in the blank treatment was higher than that of Cr(VI) treatment (see Figure 8C,D). We found that daily hatch time decreased with the increase of exposure time for the first month, and increased for the second month.

#### 3.5.2. Parental Cr(VI) Treatment Delayed Hatch Time

The hatch time of offspring treated by Cr(VI) (con-cr) was significantly shorter than that of a normal embryos (con-con) (see Figure 8E). The parental treatment (cr1/2-con&cr1/2-cr) would delay the hatching time significantly and there was no significant difference for different treatment time. The hatch time delay was found in toxicological studies of other heavy metals such as Hg [23]. Studies have shown that metal ions produced by oxidizing metals could delay the hatch time of embryos, which may be due to the effect of heavy metals on mitosis or the inhibition of the formation of embryos at a specific stage [24,25].

It is worth noting that for parental treatment, offspring in the blank (cr1/2-con) environment showed a relatively longer hatch time. We also found that there was an extremely significant interaction between parental treatment and offspring treatment on the hatch time (see Figure 8F).

#### 3.5.3. Parental Cr(VI) Treatment Increase Hatch Delay Rate

**Figure 9:**
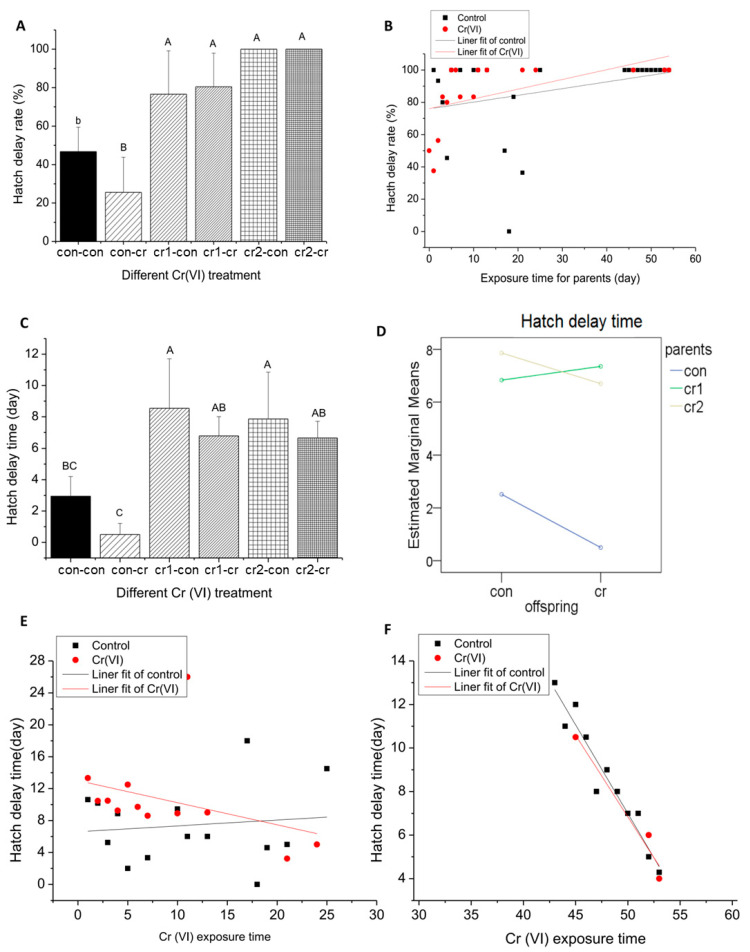
The hatch delay situation after long-term Cr(VI) exposure in marine medaka offspring. (**A**,**B**) hatch delay rate; (**A**) the hatch delay rate of offspring under different Cr(VI) treatment, different letter means the significant differences among different treatment (upper letter: *p* ≤ 0.01, lower letter: *p* ≤ 0.05); (**B**) the daily hatch delay rate under different offspring Cr(VI) exposure after parental Cr(VI) exposure, X axis represents parental treatment, and Y axis represents daily hatch delay rate, the blank line represents offspring in control environment, the red line represents offspring in Cr(VI) environment; the function relationships between exposure time and hatch delay rate were y = 0.41767x + 75.92824, R^2^ = 0.06479 (untreated offspring) and y = 0.60299x + 76.06081, R^2^ = 0.2451 (treated offspring); (**C**–**F**) hatch delay time; (**C**) the hatch delay time of offspring under different Cr(VI) treatment, different upper case means the significant differences among different treatment (*p* ≤ 0.01); different letter means the significant differences among different treatment (upper letter: *p* ≤ 0.01, lower letter: *p* ≤ 0.05) (**D**) the interaction of parent and offspring Cr(VI) treatment for hatch delay time (*p* = 0.028); (**C**,**D**), daily hatch delay time under different offspring Cr(VI) exposure after parental Cr(VI) exposure; (**E**) parental Cr(VI) exposure for first month, the function relationships between exposure time and hatch delay time were y = 007303x + 6.60125, R^2^ = −0.0685 (untreated offspring) and y = −0.27692x + 13.00838, R^2^ = 0.04457 (treated offspring); (**F**) parental Cr(VI) exposure for 2nd month, the function relationships between exposure time and hatch rate were y = −0.81409x + 47.69364, R^2^ = 0.92974 (untreated offspring) and y =−0.75x + 44.33333, R^2^ = 0.92857 (treated offspring). The number of biological samples used for each group is as follow: con-con (*N* = 297), con-cr (*N* = 34), cr1-con (*N* = 70), cr1-cr (*N* = 70), cr2-con (*N* = 28), cr2-cr (*N* = 8).

After offspring treatment (con-cr), the hatch delay rate and hatch delay time decreased. But parental Cr (VI) treatment (cr1/2-con&cr1/2-cr) resulted in a significant increase in hatching delay rate and hatch delay time (see Figure 9A,C). After parental Cr(VI) treatment, the daily hatch delay rate increased with the increase of exposure time, while daily hatch delay time kept stable for the first month and decreased dramatically for the second month (see Figure 9E,F). There was an extreme significant interaction between the parental treatment and offspring treatment in hatch delay time (see Figure 9D). It is worth noting that the hatch delay rate could reach 100% at the late stage of parental treatment.

## 4. Discussion

### 4.1. Cr(VI) Induced Reproductive Compensation Mechanism

In this study, we found that for the first month of parental Cr(VI) treatment, fish spawning was not affected, death rate and malformation rate of offspring were greatly increased and hatch rate was greatly reduced. For the second month of parental Cr(VI) treatment, fish spawning decreased obviously, the death rate and malformation rate of offspring were decreased, but hatch rate was increased. We thought that this phenomenon was a reproductive compensation mechanism for marine medaka in the Cr(VI) environment [26,27].

Organisms need to balance costs and benefits in reproductive input to achieve a higher reproductive yield [28]. From the reproductive cost hypothesis, the current reproduction have a potential trade-off effect on future reproduction [29,30]. The ultimate purpose of phenotypic plasticity induced by Cr (VI) is the optimization of reproductive input to cause specific trade-offs, including the number of offspring, survival situation, etc., [31].

Unaffected fecundity, high mortality and low hatch rate during the beginning stages of an adverse environment was found in other toxicological studies [32]. Cr (VI) was found to be neurotoxic and to interfere with cellular metabolic activity in larvae. In adult Zebrafish, no obvious neurotoxic and cellular metabolic activity was found [33]. Adult zebrafish with diet contaminated with chromium have lower viability of progeny [8]. Cr (VI) was also found to impair the pituitary-ovarian axis of a teleost Channa punctatus. Size of ovary was reduced as well as the gonadosomatic index declined [34]. This work was the first time the reproductive compensation mechanism at the late stages of an adverse environment was reported in fish toxicological studies. The reproductive compensation mechanism was important for the survival of population in adverse environment. The detail molecular mechanisms of such phenomenon need further study.

### 4.2. Parental Effects Were Stronger than Developmental Exposures

Based on one month of parental Cr(VI) exposure, we found that there were similarities and differences between parental effects and the developmental exposures, with the parental effect had a more profound influence. On the one hand, parental effects and developmental exposures displayed similar impacts on fish, such as increased death rate, increased malformation rate, and reduced hatch rate. But parental effects were often stronger than the developmental exposures, showing a greater impact.

On the other hand, there were differences between parental effects and developmental exposures. First, offspring treatment led to a reduction in hatch time, while parental treatment delayed the hatch time considerably. Second, offspring treatment only led to higher early heart rate, but parental treatment significantly reduced the overall heart rate, especially the early heart rate. These results suggests that developmental exposures would promote offspring development, whereas parental effects would delay offspring development. Different treatment of Cr(VI) have different effects on marine medaka, and the delayed development of organism can have more serious impacts.

Parental effects were stronger than the developmental exposures and the difference between the two effects may be due to the following reasons. First, parent can deliver effects to the offspring through germ cells. In the stages of embryogenesis and early life, the effects from parental generations on morphology, behavior, growth and life history can have lasting influences. Maternal transfer could also be a reason why parental exposures can affect growth of their offspring. Studies in trout and mice found that PCB is associated with lipoprotein classes and can be transferred into their offspring [35,36]. On the other hand, the absorption of pollutants by embryos is one of the important factors affecting pollutant toxic effects [37,38]. Studies on salmon eggs showed that 1 mg/L of Cr were very slightly toxic to ova compared to iron and became toxic at a concentration of 0.073 mg/L [39]. Another study showed that Cr at the concentrations from 11 to 266 μg/L did not affect the fertilization of salmon eggs [40]. The hardened egg shell of the marine medaka eggs can prevent the contaminants from entering into the embryo to reduce developmental exposures.

Traditional toxicological studies mostly focus on exposures of current generation. We found that parental effects can cause more serious effects. Therefore, transgenerational effects should be an important part for toxicological studies, which would be a valuable reference for the evaluation of pollutants.

### 4.3. Longterm Exposure of Cr(VI) Induced Adaptation

#### 4.3.1. Adaptation in the Same Generation

In this study, we found that marine medaka exposed to long-term Cr(VI) exposure might improve the survival rate of fish in the next generation. For the first month of parental exposure, offspring showed severe stress responses, such as high mortality, high malformation rate, low hatchability, low heart rate, and so on. For the second month of parental exposure, most parameters of the offspring recovered to relatively normal levels, including mortality, malformation rate, heart rate, and hatching rate. It was worth noting that the adaptation of the same generation was particularly obvious on the hatch delay time of the offspring. The hatch delay time for the first month of parental exposure stayed at a high level, while for the second month it had a straight downward trend, regardless of whether the offspring was treated by Cr(VI) or not.

This adaptive mechanism was to maintain the fitness of the organism in an adverse environment through the apparent plasticity of physiological, morphological, and behavioral responses within a short period of time [41]. Most of the environmental adaptations that involve phenotypic plasticity to cope with environmental changes can occur within one generation [42].

#### 4.3.2. Improved Adaptation in the Next Generation

We found that environmental adaptation also occurred across generations. When the parent was treated by with Cr(VI), the offspring in the blank environment (cr1/2-con) displayed more severe stress responses, such as higher mortality, higher malformation rate, lower hatch rate, and lower overall heart rate, than those in the same Cr(VI) environment (cr1/2-cr), especially for second month exposure.

We also observed adaptation on the daily changes of malformation rate, hatch rate, death rate, and hatch delay time after parental Cr(VI) exposure. On the one hand, the daily malformation rate of the offspring exposed to Cr (VI) was higher than that in the Cr(VI) environment, and the daily hatching rate was lower than the Cr(VI) environment. On the other hand, for the second month of exposure, offspring in the blank environment shows higher death rate and longer hatch delay time compared to offspring in Cr(VI) environment.

Cr(VI) could induce adaptation in the next generation [43,44]. Adaptation could be of genetic or non-genetic mechanisms. Fish with alleles that are more resistant to the Cr(VI) exposure are more likely to spawn eggs, thus passing the alleles to next generations. Non-genetic mechanisms such as epigenetic modification can pass the information from ancestor to progeny without changing the DNA sequence, but can still affect the phenotypes of offspring [45,46].

In addition, the interaction between parental and developmental exposures was related to transgenerational effects, which were significant to death rate, heart rate, hatch rate, hatch time, and hatch delay time. This interaction was more significant at the second-month exposure, resulting in a significant decrease in cumulative effect.

This is the first time that the adaptive responses of the same generation and different generations were proposed from a long time observation in the fish toxicological studies. Many studies have shown that organisms exposed to pollutants would increase stress tolerance in the offspring without exposure to pollutants [47]. Adaptive responses under different Cr(VI) environment or other adverse environments and the mechanisms of adaptive response need further study.

## 5. Conclusions

In this study, we found that Cr(VI) exposure greatly reduced female fecundity, but had no significant effect on fertilization rate. The transgenerational effects of Cr (VI) on marine medaka were manifested in malformation, death, hatching, heart development, etc.

After Cr(VI) exposure, there was a reproductive compensation mechanism for marine medaka. The high energy consumption reproduction mode was transformed into efficient reproduction mode. There were similarities and differences between parental effects and developmental exposures, but parental effects were stronger than developmental exposures. We found that the interaction between parental treatment and offspring treatment enabled the offspring to alleviate parental effect. This effect was particularly evident in the later stage of exposure. In addition, adaptation was also found in the same generation to improve the fitness in the adverse environment.

## Figures and Tables

**Table 1:** Samples for studying transgenerational effects of Cr(VI).

Sample	Cr(VI) Treatment			
	Parents		Offspring	
	Concentration	Time	Concentration	Time
con-con	0	0	0	0
con-cr	0	0	2.61 mg/L	From fertilization to hatch
cr-con1	2.61 mg/L	0–1 month	0	0
cr-cr1	2.61 mg/L	0–1 month	2.61 mg/L	From fertilization to hatch
cr-con2	2.61 mg/L	1–2 month	0	0
cr-cr2	2.61 mg/L	1–2 month	2.61 mg/L	From fertilization to hatch

## Data Availability

Data related to the study is available in the supplementary tables.

## Supplementary Materials

The following are available online at https://www.mdpi.com/2218-273X/11/2/138/s1, Table 1: Descriptives of parameters used in this study.
